# Different clinicopathologic features and favorable outcomes of patients with stage III left-sided colon cancer

**DOI:** 10.1186/s12957-015-0640-4

**Published:** 2015-08-28

**Authors:** Ching-Wen Huang, Hsiang-Lin Tsai, Ming-Yii Huang, Chun-Ming Huang, Yung-Sung Yeh, Cheng-Jen Ma, Jaw-Yuan Wang

**Affiliations:** Graduate Institute of Medicine, College of Medicine, Kaohsiung Medical University, Kaohsiung, Taiwan; Department of Surgery, Kaohsiung Municipal Hsiao-Kang Hospital, Kaohsiung Medical University, Kaohsiung, Taiwan; Division of Gastroenterology and General Surgery, Department of Surgery, Kaohsiung Medical University Hospital, Kaohsiung Medical University, Kaohsiung, Taiwan; Cancer Center, Kaohsiung Medical University Hospital, Kaohsiung Medical University, Kaohsiung, Taiwan; Division of General Surgery Medicine, Department of Surgery, Kaohsiung Medical University Hospital, Kaohsiung Medical University, Kaohsiung, Taiwan; Program of Bachelor of Health Beauty, School of Medical and Health Sciences, Fooyin University, Kaohsiung, Taiwan; Department of Radiation Oncology, Kaohsiung Medical University Hospital, Kaohsiung Medical University, Kaohsiung, Taiwan; Division of Trauma, Department of Surgery, Kaohsiung Medical University Hospital, Kaohsiung Medical University, Kaohsiung, Taiwan; Graduate Institute of Clinical Medicine, College of Medicine, Kaohsiung Medical University, Kaohsiung, Taiwan; Department of Surgery, Faculty of Medicine, College of Medicine, Kaohsiung Medical University, Kaohsiung, Taiwan

**Keywords:** Right-sided colon, Left-sided colon, Stage III, Overall survival, Cancer-specific survival

## Abstract

**Background:**

We evaluated the clinicopathologic features and outcomes of Taiwanese patients with right-sided versus left-sided colon cancer according to various cancer stages.

**Methods:**

A total of 1095 patients with primary colorectal cancer (CRC) undergoing surgery at a single-institution were enrolled. We analyzed patient differences in terms of clinicopathologic features, overall survival (OS) and cancer-specific survival (CSS) of right- versus left-sided colon cancer.

**Results:**

Right-sided colon cancers were noted in 249 (22.7 %) patients, and left-sided colon cancers were noted in 846 (77.3 %) patients. Right-sided colon cancers were found to be significantly larger (*P* = 0.003) and poorly differentiated (*P* < 0.001), while also exhibiting advanced depth of tumor invasion (*P* = 0.002) and advanced UICC/AJCC stage (*P* = 0.016). Patients with right-sided colon cancers had both poorer OS and CSS than those with left-sided colon cancers (*P* = 0.021 and 0.023, respectively). However, analysis by various stages revealed significant OS and CSS differences (*P* = 0.002 and 0.002, respectively) between right-sided and left-sided colon cancers only in stage III patients.

**Conclusions:**

This study demonstrated poorer OS and CSS in patients with right-sided versus those with left-sided colon cancers, but significant differences were noted only in stage III patients.

## Background

Colorectal cancer (CRC) is the third most common cancer and the third leading cause of cancer death in the United States, where an estimated 142,820 newly diagnosed cases of CRC and an estimated 50,830 cancer deaths from CRC were reported in 2013 [1]. In Taiwan, CRC is the most common cancer type, having increased rapidly in prevalence in recent years and was third leading cause of cancer-related death as of 2012. The incidence of CRC was 32.38 per 100,000 (7,213 new diagnoses of CRC) in 2000 and 60.72 per 100,000 (14,040 new diagnoses of CRC) in 2010, and in 2012, 5131 people died from CRC, resulting in a death rate of 22.0 per 100,000 [2].

There were various embryological and biological differences between the proximal and distal colon. The proximal segment of the large intestine, including the cecum, ascending colon, and proximal two-thirds of the transverse colon, arises from embryonic midgut and receives blood perfusion from the superior mesentery artery. The distal segment of the large intestine, from the splenic flexure to the upper anal canal, arises from embryonic hindgut and receives blood perfusion from the inferior mesentery artery. Bufill [3] reported that there were differences in the epidemiologic, pathologic, cytogenetic, and molecular features of proximal and distal CRC, as well as indications of differing carcinogenetic mechanisms. Right-sided colon cancers tend to be bulky, exophytic, polypoid lesions growing into the colon lumen and causing anemia. However, left-sided colon cancers tend to be infiltrating, constricting lesions encircling the colorectal lumen and causing obstruction. Sex and age disparities have also been reported [4, 5], and different clinicopathologic features of the right colon and left colon have likewise been noted. Meguid et al. [6] reported a higher proportion of poorly differentiated tumors, larger tumors, and stage III disease in right-sided colon cancers than in left-sided colon cancers. Similar results have also been presented in other studies [7–10].

In addition to different clinicopathologic features, there are genetic differences between the right colon and left colon. Microsatellite instability (MSI) and chromosomal instability (CIN) are the two most frequently mentioned of these genetic differences. MSI-high tumors have been reported to be more frequent in the right colon [11–13]. Nevertheless, CIN has been reported to be more frequent in the left colon [11–13]. MSI-high tumors have also been shown to present aggressive histological types, such as poor differentiation.

In spite of the aforementioned clinicopathologic and genetic differences, there are still controversies regarding differences in the clinical outcomes of patients with right colon and left colon [6–10, 14–19]. In order to help resolve these controversies, we conducted a retrospective study to evaluate the clinicopathologic features and clinical outcomes of Taiwanese colorectal cancer (CRC) patients with right colon cancer versus those with left colon cancer according to the various cancer stages.

## Methods

### Patients

There were 1198 consecutive patients with histologically proven CRC (adenocarcinoma) who received surgical treatment with curative intent from a single-institution- Kaohsiung Medical University Hospital from January 2002 to December 2008. Twenty-two patients without complete follow-up data were excluded. Moreover, eighty-one patients with second cancers were also excluded. In total, then, this retrospective study included 1095 patients with CRC. The present study was approved by the Institutional Review Board of the Kaohsiung Medical University Hospital. Patients’ clinical outcomes and survival statuses were regularly followed up. Available variables included the following: age at diagnosis, sex, tumor size, histological type, TNM classification, vascular invasion, perineural invasion, preoperative serum level of albumin, preoperative and postoperative serum level of CEA (Carcinoembryonic antigen), comorbidity of diabetes mellitus (DM), comorbidity of cardiac disease, comorbidity of renal disease, whether or not the patient received chemotherapy, and body mass index (BMI). Right-sided colon cancers were defined as those located in the cecum, ascending colon, hepatic flexure, and transverse colon, while left-sided cancers were defined as those located in the splenic flexure, descending colon, sigmoid, and rectum. This definition was similar to previous studies [4, 5, 13, 20–22]. Diagnoses of DM were made according to chart records indicating a history of DM or the use of medicines for DM. Preoperative serum levels of albumin and CEA were checked within 1 week before the operation, and postoperative serum levels of CEA were checked at least 4 weeks after. The cut-off values of serum albumin and CEA were set at 3.5 gm/dl and 5 ng/ml, respectively. The existence of comorbidity was determined by chart record according to the International Classification of Diseases (ICD, 9^th^ version), that is 390 ~ 398, 410 ~ 414, and 420 ~ 429 for cardiac disease, and 584 ~ 588 for renal disease. The TNM classification was defined according to the criteria of the American Joint Commission on Cancer/International Union Against Cancer (AJCC/UICC) [23]. All patients were followed up until their deaths, their last follow-up, or December 31, 2010. The median follow-up time was 37 months (range: 4–97 months). Overall survival (OS) was defined as the time from the date of primary treatment to the date of death from any cause or until the date of the last follow-up. Cancer-specific survival was defined as the time from primary surgery and death from CRC or until the date of the last follow-up.

### Statistical analysis

All data were statistically analyzed using the Statistical Package for the Social Sciences, version 19.0 (SPSS Inc., Chicago, IL, USA). The correlation between clinicopathological features and tumor location (right-sided colon vs left-sided colon) was calculated using a Chi-square test (for categorical variables) and Student t-test (for continuous variables). The Cox proportional-hazards model was used for univariate and multivariate analyses to identify the independent prognostic factors for OS and CSS. OS and CSS were calculated by the Kaplan-Meier method, and the differences in survival rates were analyzed by the log-rank test. A *P* value of less than 0.05 was considered to be statistically significant.

## Results

### Characteristics of patients at all stages

The clinical and pathologic data regarding all 1095 CRC patients are summarized in Table 1. Of the 1095 patients, 249 (22.7 %) had right-sided colon cancers, including cecal cancers (1.5 %), ascending colon cancers (13.9 %), and transverse colon cancers (7.4 %), and 846 (77.3 %) had left-sided colon cancers, including descending colon cancers (9.2 %), sigmoid colon cancers (34.9 %), and rectal cancers (33.2 %). The proportion of tumors with sizes ≥ 5 cm was significant higher and poorly differentiated cancers were more common in patients with right-sided colon cancers than in those with left-sided colon cancers. Meanwhile, locally advanced cancers were also more common in patients with right-sided colon cancers than in those with left-sided colon cancers (T3 + T4: 85.5 % vs 76.5 %, *P* = 0.017). Patients with right-sided colon cancers had less stage I CRC and more stage II and III CRC (*P* = 0.016) than those with left-sided colon cancers. Higher postoperative serum CEA levels were more frequently observed in patients with right-sided colon cancers than in those with left-sided colon cancers. However, there were no significant differences found in terms of age at diagnosis, gender, lymph node metastasis, vascular invasion, perineural invasion, pre-operative serum CEA level, pre-operative serum albumin level, the percentages of patients with diabetes mellitus, the percentages of patients receiving chemotherapy, the frequencies of cardiac disease comorbidity, the frequencies of renal disease comorbidity, or BMI. There is no difference in the quality of the operations for the left-sided colon and right-sided colon in this study (data not shown).

**Table 1:** Baseline characteristics of 1095 colorectal cancer patients by tumor location

Characteristic	Right colon (%)	Left colon (%)	*P* value
	N = 249 (22.7 %)	N = 846 (77.3 %)	
Age (years, mean ± SD)	64.94 ± 13.49	63.82 ± 12.85	0.233
Gender			0.118
Female	119 (47.8)	357 (42.2)	
Male	130 (52.2)	489 (57.8)	
Tumor size			0.003*
< 5 cm	121 (48.8)	495 (59.4)	
≥ 5 cm	127 (51.2)	339 (40.6)	
Histology			<0.001*
Well	20 (8.2)	51 (6.2)	
Moderately	181 (74.5)	704 (85.7)	
Poorly	42 (17.3)	66 (8.0)	
AJCC Stage^a^ (Initial diagnosis)			0.016*
I	28 (11.2)	164 (19.4)	
II	101 (40.6)	286 (33.8)	
III	80 (32.1)	251 (29.7)	
IV	40 (16.1)	145 (17.1)	
Tumor depth			0.017*
T1	12 (4.8)	59 (7.0)	
T2	24 (9.7)	139 (16.5)	
T3	199 (80.3)	591 (70.4)	
T4	13 (5.2)	51 (6.1)	
Lymph Node metastasis			0.677
N0	142 (57.3)	491 (58.7)	
N1	62 (25.0)	217 (25.9)	
N2	44 (17.7)	129 (15.4)	
Lymph Node metastasis			0.694
No	142 (57.3)	491 (58.7)	
Yes	106 (42.7)	346 (41.3)	
Vascular invasion			0.529
No	163 (66.5)	561 (68.7)	
Yes	82 (33.5)	256 (31.3)	
Perineural invasion			0.833
No	157 (63.8)	516 (63.1)	
Yes	89 (36.2)	302 (36.9)	
Pre-op serum CEA^b^ level			0.496
< 5 ng/ml	121 (51.5)	424 (54.0)	
≥ 5 ng/ml	114 (48.5)	361 (46.0)	
Post-op serum CEA^b^ level			0.023*
< 5 ng/ml	158 (68.1)	594 (75.6)	
≥ 5 ng/ml	74 (31.9)	192 (24.4)	
Pre-op serum Albumin level (g/dl)	3.56 ± 0.44	3.63 ± 0.45	0.062
Diabetes mellitus			0.731
Yes	60 (24.1)	195 (23.0)	
No	189 (75.9)	651 (77.0)	
Chemotherapy			0.427
Yes	161 (64.9)	570 (67.6)	
No	87 (35.1)	273 (32.4)	
Cardiac disease			0.764
Yes	103 (41.4)	359 (42.4)	
No	146 (58.6)	487 (57.6)	
Renal disease			0.690
Yes	13 (5.2)	39 (4.6)	
No	236 (94.8)	807 (95.4)	
BMI^c^	23.53 ± 4.25	23.63 ± 3.62	0.758

^a^AJCC American Joint Commission on Cancer

^b^CEA Carcinoembryonic antigen

^c^BMI Body mass index

*Indicated *P* < 0.05

### Univariate and multivariable analyses of Overall Survival (OS) and Cancer-specific Curvival (CSS) for patients at all stages

Univariate and multivariate analyses were performed to investigate independent prognostic factors for OS and CSS using the Cox proportional-hazards model (Table 2). Old age, poor differentiation, vascular invasion, perineural invasion, post-operative serum CEA level, and comorbidity of renal disease were demonstrated to be independent negative prognostic factors for OS and CSS.

**Table 2:** Univariate and multivariable analysis of prognostic indicators on overall survival and cancer-specific survival for 1095 colorectal cancer patients

Parameters	Overall survival				Cancer-specific survival			
	Univariate analysis		Multivariable analysis		Univariate analysis		Multivariable analysis	
	HR^d^ (95 % CI^e^)	*P* value	HR^d^ (95 % CI^e^)	*P* value	HR^d^ (95 % CI^e^)	*P* value	HR^d^ (95 % CI^e^)	*P* value
Age (years)	1.352 (1.099 – 1.664)	0.002*	1.347 (1.008 – 1.801)	0.044*	1.349 (1.096 – 1.601)	0.005*	1.362 (1.018 – 1.822)	0.037*
≥65 vs <65 (576/519)								
Gender	1.098 (0.893 – 1.349)	0.377	1.036 (0.795 – 1.506)	0.792	1.096 (0.891 – 1.347)	0.386	1.032 (0.792 – 1.345)	0.817
Male vs Female (619/476)								
Location	1.309 (1.041 – 1.647)	0.021*	1.172 (0.870 – 1.580)	0.296	1.303 (1.036 – 1.639)	0.024*	1.168 (0.867 – 1.572)	0.308
Right vs Left (249/846)								
Tumor size	1.477 (1.200 – 1.817)	<0.001*	0.981 (0.745 – 1.292)	0.893	1.480 (1.203 – 1.821)	<0.001*	0.979 (0.744 – 1.289)	0.880
≥5 cm vs <5 cm (466/612)								
Tumor depth	2.959 (2.088 – 4.193)	<0.001*	1.385 (0.596 – 3.220)	0.449	2.867 (2.094 – 4.205)	<0.001*	1.379 (0.593 – 3.205)	0.455
T3 + T4 vs T1 + T2 (853/235)								
Lymph Node metastasis	2.464 (1.996 – 3.042)	<0.001*	1.055 (0.644 – 1.730)	0.831	2.460 (1.992 – 3.037)	<0.001*	1.060 (0.647 – 1.738)	0.816
Yes vs No (452/633)								
AJCC^a^ stage	3.441 (2.753 – 4.302)	<0.001*	1.375 (1.032 – 1.760)	0.015*	3.436 (2.748 – 4.295)	<0.001*	2.022 (1.065-2.099)	<0.001*
III + IV vs I + II (516/579)								
Histology	1.997 (1.510 – 2.642)	<0.001*	1.655 (1.153 – 2.275)	0.006*	2.012 (1.521 – 2.662)	<0.001*	1.687 (1.177 – 2.419)	0.004*
PD vs MD + WD^b^ (108/952)								
Vascular invasion	2.796 (2.261 – 3.457)	<0.001*	1.642 (1.228 – 2.149)	0.001*	2.783 (2.251 – 3.441)	<0.001*	1.623 (1.226 – 2.418)	0.001*
Yes vs No (338/724)								
Perineurial invasion	2.437 (1.971 – 3.013)	<0.001*	1.787 (1.358 – 2.351)	<0.001*	2.423 (1.960 – 2.996)	<0.001*	1.765 (1.341 – 2.322)	<0.001*
Yes vs No (391/673)								
Pre-op CEA^c^ (ng/ml)	3.047 (2.423 – 3.832)	<0.001*	1.249 (0.902 – 1.729)	0.180	3.053 (2.428 -–3.840)	<0.001*	1.245 (0.899 – 1.723)	0.186
≥5/ vs <5 (475/545)								
Post-op CEA ^c^ (ng/ml)	5.567 (4.456 – 6.956)	<0.001*	2.492 (1.817 – 3.418)	<0.001*	5.591 (4.474 – 6.986)	<0.001*	2.514 (1.832 – 3.449)	<0.001*
≥5 vs <5 (266/752)								
Pre-op albumin (g/dl)	1.847 (1.474 – 2.314)	<0.001*	1.245 (0.933 – 1.662)	0.137	1.828 (1.460 – 2.290)	<0.001*	1.221 (0.915 – 1.630)	0.175
<3.5 vs ≥3.5 (324/572)								
Diabetes mellitus	1.012 (0.795 – 1.288)	0.923	0.962 (0.710 – 1.305)	0.804	1.012 (0.795 – 1.288)	0.924	0.949 (0.700 – 1.288)	0.738
Yes vs No (255/840)								
Cardiac disease	1.162 (0.947 – 1.427)	0.150	1.016 (0.772 – 1.337)	0.909	1.162 (0.947 – 1.426)	0.151	1.019 (0.774 – 1.342)	0.895
Yes vs No (462/633)								
Renal disease	2.583 (1.777 – 3.754)	<0.001*	3.139 (1.854 – 5.317)	<0.001*	2.588 (1.780 – 3.761)	<0.001*	3.101 (1.830 – 5.254)	<0.001*
Yes vs No (52/1043)								
Chemotherapy	1.509 (1.188 – 1.916)	<0.001*	1.233 (0.831 – 1.828)	0.298	1.580 (1.188 – 1.915)	0.001*	1.230 (0.830 – 1.824)	0.302
Yes vs No (731/360)								

^a^AJCC American Joint Commission on Cancer, ^b^PD Poorly differentiated, MD Moderately differentiated, WD Well differentiated

^c^CEA Carcinoembryonic antigen, ^d^HR Hazard ratio, ^e^Confidence interval

*Indicated *P* < 0.05

**Fig. 1:**
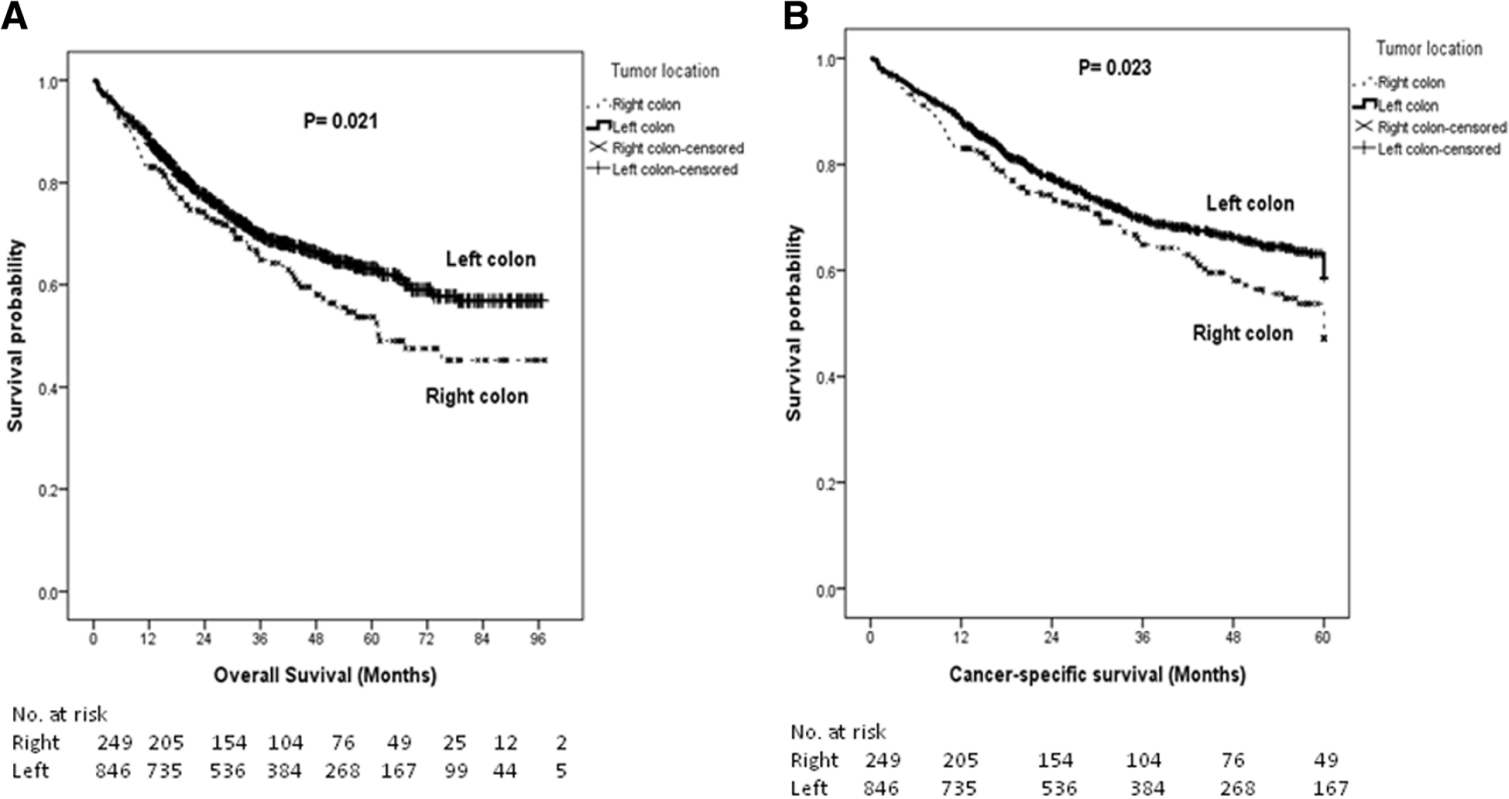
The Kaplan-Meier survival curves for patients with right- and left-sided colon cancers (all stages combined). **a** Overall survival; (**b**) Cancer-specific survival

The Kaplan-Meier survival analysis demonstrated that patients with right-sided colon cancer had poorer OS (*P* = 0.021) and CSS (*P* = 0.023) than patients with left-sided colon cancer (Fig. 1a and b). The median OS times of patients with right-sided colon cancer and those with left-sided colon cancer were 60.33 and 67.37 months (*P* = 0.021; 95 % CI, 54.982-65.679 and 64.528-70.204), respectively. The 5-year OS rates of patients with right-sided colon cancer and those with left-sided colon cancer were 47 % and 59 %, respectively. The median CSS times of patients with right-sided colon cancer and those with left-sided colon cancer were 42.94 and 45.88 months (*P* = 0.023; 95 % CI, 40.114-45.765 and 44.412-47.346), respectively. The 5-year CSS rates of patients with right-sided colon cancer and those with left-sided colon cancer were 42 % and 55 %, respectively.

### Characteristics of 331 patients with stage III disease

Subgroup analyses by stage revealed significant differences in terms of OS and CSS only in patients with stage III CRC, but not in patients with stages I, II, or IV CRC. The clinical and pathological data were analyzed by stage and are summarized in Table 3. Of the 331 patients with stage III CRC, eighty patients (24.2 %) were right-sided colon cancers and 251 patients (75.8 %) were left-sided colon cancers. Poorly differentiated cancers were more common and the proportion of tumors with sizes ≥ 5 cm was higher in patients with right-sided colon cancers than in those with left-sided colon cancers. Higher pre-operative and postoperative serum CEA levels were more frequently observed in patients with right-sided colon cancers than in those with left-sided colon cancers. However, there were no significant differences found in terms of age at diagnosis, gender, tumor depth, lymph node metastasis, vascular invasion, perineural invasion, pre-operative serum albumin level, the percentages of patients with diabetes mellitus, the percentages of patients receiving chemotherapy, the frequencies of cardiac disease comorbidity, the frequencies of renal disease comorbidity, or BMI.

**Table 3:** Baseline characteristics of 1095 colorectal cancer patients stratified by stage and tumor location

Characteristic	Stage I			Stage II			Stage III			Stage IV		
	Right-sided colon (%)	Left-sided colon (%)	*P* value	Right-sided colon (%)	Left-sided colon (%)	*P* value	Right-sided colon (%)	Left-sided colon (%)	*P* value	Right-sided colon (%)	Left-sided colon (%)	*P* value
	N = 28 (14.6 %)	N = 164 (85.3 %)		N = 101 (26.1 %)	N = 286 (73.9 %)		N = 80 (24.2 %)	N = 251 (75.8 %)		N = 40 (21.4 %)	N = 145 (78.4 %)	
Age (years, mean ± SD)	67.36 ± 10.05	65.04 ± 13.20	0.289	65.90 ± 13.67	65.30 ± 12.15	0.678	61.99 ± 14.86	61.90 ± 13.21	0.959	66.70 ± 11.57	62.85 ± 12.77	0.087
Gender			0.603			0.244			0.751			0.011*
Female	12 (42.9)	79 (48.2)		52 (47.3)	128 (40.9)		37 (46.3)	111 (44.2)		23 (57.5)	51 (35.2)	
Male	16 (57.1)	85 (51.8)		58 (52.7)	185 (59.1)		43 (53.8)	140 (55.8)		17 (42.5)	94 (64.8)	
Tumor size			1.000			0.060			0.070			0.921
< 5 cm	24 (85.7)	139 (85.8)		42 (41.6)	150 (52.4)		37 (46.3)	144 (57.8)		18 (46.2)	62 (45.3)	
≥ 5 cm	4 (14.3)	23 (14.2)		59 (58.4)	136 (47.6)		43 (53.8)	105 (42.2)		21 (53.8)	75 (54.7)	
Histology			0.195			0.002*			0.036*			0.517
Well	10 (35.7)	32 (20.3)		6 (6.1)	11 (3.9)		3 (3.9)	5 (2.0)		1 (2.6)	3 (2.2)	
Moderately	17 (60.7)	120 (75.9)		76 (76.8)	255 (90.1)		57 (74.0)	212 (86.5)		31 (79.5)	117 (86.7)	
Poorly	1 (3.6)	6 (3.8)		17 (17.2)	17 (6.0)		17 (22.1)	28 (11.4)		7 (17.9)	15 (11.1)	
Tumor depth			0.872			0.197			0.457			0.858
T1	10 (35.7)	56 (34.1)		0 (0.0)	0 (0.0)		2 (2.5)	2 (0.8)		0 (0.0)	1 (0.7)	
T2	18 (64.3)	108 (65.9)		0 (0.0)	0 (0.0)		5 (6.3)	25 (10.0)		1 (2.6)	6 (4.3)	
T3	0 (0.0)	0 (0.0)		101 (100.0)	279 (97.6)		67 (83.8)	209 (83.3)		31 (79.5)	103 (74.1)	
T4	0 (0.0)	0 (0.0)		0 (0.0)	7 (2.4)		6 (7.5)	15 (6.0)		7 (17.9)	29 (20.9)	
Lymph Node metastasis									0.158			0.813
N0	0 (0.0)	0 (0.0)		0 (0.0)	0 (0.0)		0 (0.0)	0 (0.0)		13 (33.3)	41 (30.1)	
N1	0 (0.0)	0 (0.0)		0 (0.0)	0 (0.0)		49 (61.3)	175 (69.7)		13 (33.3)	42 (30.9)	
N2	0 (0.0)	0 (0.0)		0 (0.0)	0 (0.0)		31 (38.8)	76 (30.3)		13 (33.3)	53 (39.0)	
Vascular invasion			0.695			0.133			0.110			0.510
No	27 (96.4)	146 (92.4)		83 (82.2)	211 (74.8)		39 (48.8)	145 (58.9)		14 (38.9)	59 (45.0)	
Yes	1 (3.6)	12 (7.6)		18 (17.8)	71 (25.2)		41 (51.2)	101 (41.1)		22 (61.1)	72 (55.0)	
Perineural invasion			0.473			0.861			0.512			0.790
No	27 (96.4)	143 (90.5)		67 (66.3)	185 (65.4)		47 (59.5)	136 (55.3)		16 (42.1)	52 (39.7)	
Yes	1 (3.6)	15 (9.5)		34 (33.7)	98 (34.6)		32 (40.5)	110 (44.7)		22 (57.9)	79 (60.3)	
Pre-op serum CEA^a^ level			0.560			0.237			0.043*			0.410
< 5 ng/ml	22 (81.5)	127 (85.8)		62 (62.6)	150 (55.8)		28 (38.4)	122 (51.9)		9 (25.0)	25 (18.8)	
≥ 5 ng/ml	5 (18.5)	21 (14.2)		37 (37.4)	119 (44.2)		45 (61.6)	113 (48.1)		27 (75.0)	108 (81.2)	
Post-op serum CEA^a^ level			0.697			0.158			0.025*			0.764
< 5 ng/ml	24 (96.0)	140 (92.1)		75 (79.8)	232 (85.9)		49 (64.5)	185 (77.4)		10 (27.0)	37 (29.6)	
≥ 5 ng/ml	1 (4.0)	12 (7.9)		19 (20.2)	38 (14.1)		27 (35.5)	54 (22.6)		27 (73.0)	88 (70.4)	
Pre-op serum Albumin level (g/dl)	3.72 ± 0.37	3.76 ± 0.39	0.664	3.46 ± 0.47	3.58 ± 0.46	0.030	3.68 ± 0.37	3.69 ± 0.40	0.887	3.48 ± 0.45	3.46 ± 0.50	0.885
Diabetes mellitus			0.307			0.841			0.920			0.844
Yes	9 (32.1)	38 (23.2)		24 (23.8)	69 (24.1)		19 (23.8)	61 (24.3)		8 (20.0)	27 (18.6)	
No	19 (67.9)	126 (76.8)		77 (76.2)	217 (75.9)		62 (76.2)	190 (75.7)		32 (80.0)	118 (81.4)	
Chemotherapy						0.030*			0.852			0.817
Yes				53 (52.5)	185 (64.7)		72 (91.1)	227 (90.4)		33(82.6)	121 (84.0)	
No				48 (47.5)	101 (35.3)		7 (8.9)	24 (9.6)		7 (17.5)	23 (16.0)	
Cardiac disease			0.257			0.365			0.772			0.976
Yes	15 (53.6)	69 (42.1)		41 (40.6)	131 (45.8)		32 (40.0)	105 (41.8)		15 (37.5)	54 (37.2)	
No	13 (46.4)	95 (57.9)		60 (59.4)	156 (54.2)		48 (60.0)	146 (58.2)		25 (62.5)	91 (62.8)	
Renal disease			0.127^*^			0.693			0.450			1.000
Yes	3 (10.7)	6 (3.7)		6 (5.9)	14 (4.9)		2 (2.5)	11 (4.4)		2 (5.0)	8 (5.5)	
No	25 (89.3)	158 (96.3)		95 (94.1)	272 (95.1)		78 (97.5)	240 (95.6)		38 (95.0)	137 (94.5)	
BMI^b^	23.94 ± 3.88	23.70 ± 3.73	0.748	23.80 ± 4.39	23.73 ± 3.73	0.092	23.71 ± 4.13	23.95 ± 3.44	0.616	22.23 ± 4.31	22.77 ± 3.50	0.418

^a^CEA Carcinoembryonic antigen, ^b^BMI Body mass index, *Indicated *P* < 0.05

### Univariate and multivariable analyses of Overall Survival (OS) and Cancer-specific Survival (CSS) for patients with stage III CRC

Univariate and multivariate analyses were performed to investigate independent prognostic factors for OS and CSS using the Cox proportional-hazards model (Table 4). Vascular invasion, perineural invasion, post-operative serum CEA level, and comorbidity of renal disease were demonstrated to be independent negative prognostic factors for OS and CSS.

**Table 4:** Univariate and multivariable analysis of prognostic indicators on overall survival and cancer-specific survival for 331 stage III colorectal cancer patients

Parameters	Overall survival				Cancer-specific survival			
	Univariate analysis		Multivariable analysis		Univariate analysis		Multivariable analysis	
	HR^c^ (95 % CI^d^)	*P* value	HR^c^ (95 % CI^d^)	*P* value	HR^c^ (95 % CI^d^)	*P* value	HR^c^ (95 % CI^d^)	*P* value
Age (years)	1.157 (0.801 – 1.671)	0.437	1.032 (0.607 – 1.754)	0.909	1.157(0.801 – 1.670)	0.438	1.032 (0.607 – 1.757)	0.906
≥65 vs <65 (151/180)								
Gender	1.347 (0.926 – 1.960)	0.119	1.348 (0.795 – 1.356)	0.227	1.336 (0.918 – 1.942)	0.130	1.341 (0.827 – 2.176)	0.235
Male vs Female (183/148)								
Location	1.828 (1.242 – 2.681)	0.002*	1.536 (0.939 – 2.513)	0.087	1.842 (1.253 – 2.703)	0.002*	1.548 (0.946 – 2.532)	0.082
Right vs Left (80/251)								
Tumor size	1.625 (1.120 – 2.359)	0.011	1.557 (0.951 – 2.548)	0.078	1.617 (1.114 – 2.345)	0.011*	1.544 (0.944 – 2.527)	0.084
≥5 cm vs <5 cm (148/181)								
Tumor depth	1.639 (0.799 – 3.363)	0.178	0.935 (0.347 – 2.515)	0.894	1.651 (0.805 – 3.388)	0.171	0.937 (0.348 – 2.520)	0.897
T3 + T4 vs T1 + T2 (297/34)								
Histology	2.171 (1.378 – 3.419)	0.001*	1.761 (0.969 – 3.201)	0.063	2.185 (1.387 – 3.442)	0.001*	1.767 (0.972 – 3.210)	0.062
PD vs MD + WD^a^ (45/277)								
Vascular invasion	2.307 (1.565 – 3.399)	<0.001*	1.784 (1.091 – 2.915)	0.021*	2.291 (1.555 – 3.376)	<0.001*	1.777 (1.087 – 2.904)	0.022*
Yes vs No (142/184)								
Perineural invasion	1.866 (1.286 – 2.765)	0.001*	1.822 (1.112 – 2.986)	0.017*	1.880 (1.282 – 2.757)	0.001*	1.820 (1.111 – 2.983)	0.017*
Yes vs No (142/183)								
Pre-op CEA^b^ (ng/ml)	1.925 (1.288 – 2.875)	0.001*	1.237 (0.697 – 2.195)	0.468	1.945 (1.301 – 2.908)	0.001*	1.253 (0.706 – 2.224)	0.441
≥5/ vs <5 (158/150)								
Post-op CEA^b^ (ng/ml)	3.253 (2.198 – 4.816)	<0.001*	2.053 (1.187 – 3.550)	0.010*	3.285 (2.218 – 4.864)	<0.001*	2.064 (1.193 – 3.571)	0.010*
≥5 vs <5 (81/234)								
Pre-op albumin (g/dl)	1.480 (0.965 – 2.269)	0.072	1.237 (0.740 – 2.067)	0.417	1.474 (0.961 – 2.259)	0.075	1.226 (0.734 – 2.046)	0.437
<3.5 vs ≥3.5 (85/185)								
Diabetes mellitus	1.331 (0.888 – 1.996)	0.166	1.103 (0.633 – 1.924)	0.729	1.325 (0.884 –1.987)	0.173	1.091 (0.626 – 1.903)	0.758
Yes vs No (80/251)								
Cardiac disease	1.337 (0.926 – 1.931)	0.121	0.796 (0.464 – 1.366)	0.407	1.342 (0.929 – 1.938)	0.117	0.799 (0.465 – 1.372)	0.415
Yes vs No (137/194)								
Renal disease	4.151 (2.164 – 7.963)	<0.001*	2.981 (1.089 – 8.157)	0.033*	4.165 (2.171 – 7.999)	<0.001*	2.979 (1.088 – 8.159)	0.034*
Yes vs No (13/318)								
Chemotherapy	0.437 (0.257 – 0.742)	0.002*	1.061 (0.434 – 2.594)	0.897	0.435 (0.256 – 0.738)	0.002*	1.078 (0.440 – 2.639)	0.869
Yes vs No (31/299)								

^a^PD Poorly differentiated, MD Moderately differentiated, WD Well differentiated, ^b^ CEA Carcinoembryonic antigen, ^c^HR Hazard ratio,

^d^Confidence interval

*Indicated *P* < 0.05

**Fig. 2:**
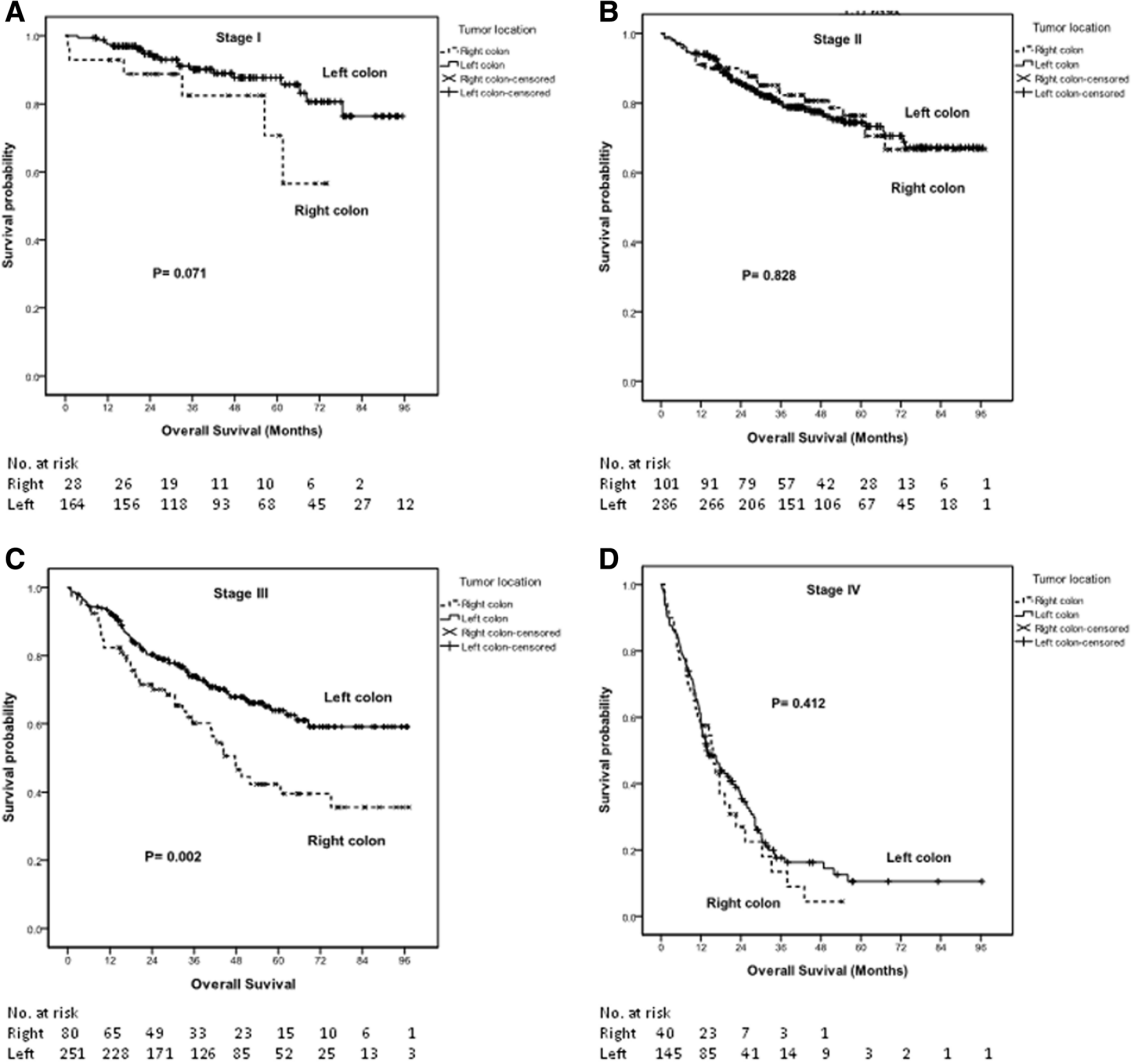
The Kaplan-Meier survival curves for overall survival of patients with right- and left-sided colon cancers (according to stage). **a** Stage I disease; (**b**) Stage II disease; (**c**) Stage III disease; and (**d**) Stage IV disease

**Fig. 3:**
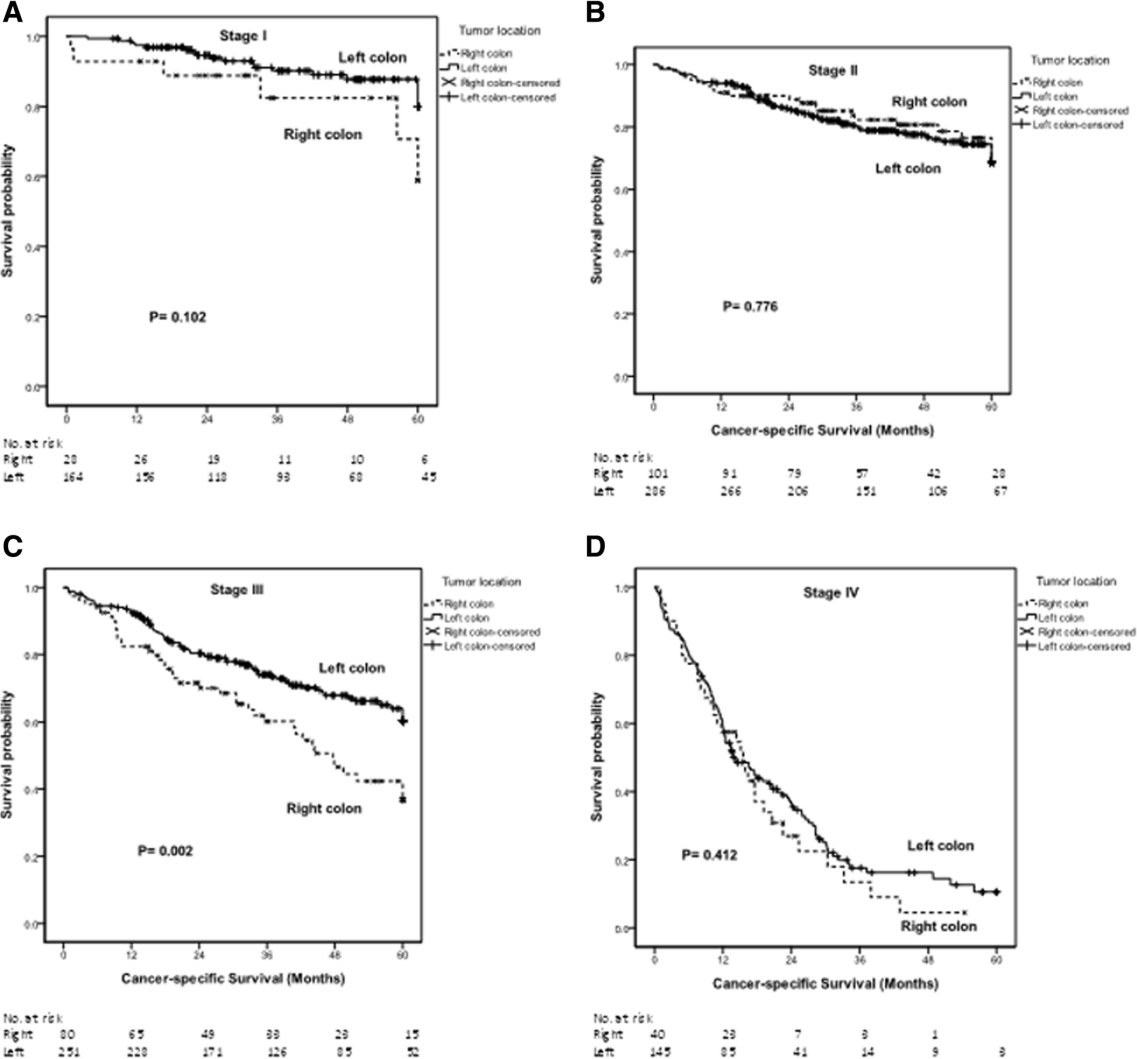
The Kaplan-Meier survival curves for cancer-specific survival of patients with right- and left-sided colon cancers (according to stage). **a** Stage I disease; (**b**) Stage II disease; (**c**) Stage III disease; and (**d**) Stage IV disease

The Kaplan-Meier survival analysis demonstrated that patients with right-sided colon cancer had poorer OS (*P* = 0.002) (Fig. 2c) and CSS (*P* = 0.002) (Fig. 3c). The median OS times of patients with right-sided colon cancer and those with left-sided colon cancer were 54.06 and 69.57 months (*P* = 0.002; 95 % CI, 45.207-62.903 and 64.505-74.633), respectively. The 5-year OS rates of patients with right-sided colon cancer and those with left-sided colon cancer were 39 % and 60 %, respectively. The median CSS times of patients with right-sided colon cancer and those with left-sided colon cancer were 40.20 and 47.53 months (*P* = 0.002; 95 % CI, 35.328-45.069 and 44.985-50.068), respectively. The 5-year CSS rates of patients with right-sided colon cancer and those with left-sided colon cancer were 32 % and 57 %, respectively.

## Discussion

In the present study, 22.7 % (249) patients had right-sided colon cancers and 77.3 % (846) patients had left-sided colon cancers. We showed that patients with right-sided colon cancer had significantly worse OS and CSS than patients with left-sided colon cancer. We also demonstrated that the differences in OS and CSS were still significant only in patients with stage III CRC.

The large intestine can be differentiated to proximal and distal part in relation to the splenic flexure because the two parts have two different embryologic origins, with differing physiological and biological characteristics [13]. On the basis of underlying genetic mutational pathways, Gervaz P et al. [13] reported that CRC could be differentiated into two phenotypes. They also emphasized the new paradigm of two colons-two cancers, which declared the connection between embryology, physiology, and molecular biology. They also suggested that location of the neoplasm in relation to the splenic flexure should be considered before group stratification in future trials of adjuvant chemotherapy. In the present study, therefore, we divided the large intestine into two segments in relation to the splenic flexure. The right-sided colon includes the cecum, ascending colon, hepatic flexure, and transverse colon, and the left-sided colon includes the splenic flexure, descending colon, sigmoid and rectum.

Recent reports have indicated an increasing percentage of right-sided colon cancers, with the percentage rising from 33 % to 67 % [5–8, 10, 13, 17–19]. However, only 22.7 % of the patients in the present study had right-sided colon cancers. Stratified by stage, 14.6 %, 26.1 %, 24.2 %, and 21.4 % of those patients had right-sided colon cancers in stages I, II, III, and IV, respectively.

Right-sided colon cancers significantly tended to be larger in the present study, consistent with previous reports [6, 9]. Higher proportions of poorly differentiated tumors were noted in right-sided colon cancers than in left-sided colon cancers in all stages of the disease, in accordance with previous studies [6–10, 17, 20]. We also found similar results in stage II disease and stage III disease. Meanwhile, local advanced tumors were more common in right-sided colon cancers than left-sided colon cancers, consistent with previous studies [7, 8, 10, 14, 19, 24]. In addition, we also demonstrated higher proportion of stage II and III diseases and a lower proportion of stage I diseases in patients with right-sided colon cancers, consistent with the result reported by Meguid et al., Benedix et al. and Weiss et al. [6–8].

The 5-year OS rate of patients with right-sided colon cancer was 12 % lower than the rate of those with left-sided colon cancer. In addition, the 5-year CSS rate of patients with right-sided colon cancer was 13 % lower than the rate of those with left-sided colon cancer. Meguid et al. [6] reported a 3.4 % lower 5-year OS rate, 4.1 % lower 10-year OS rate, and 4 % lower 15-year OS rate in patients with right-sided colon cancer compared with patients with left-sided colon cancer. Benedix et al. [7] reported a 4 % lower 5-year OS rate in patients with right-sided colon cancer and the difference in the 5-year disease-free survival rates was only marginal. Suttie et al. [14] presented a worse prognosis for patients with right-sided colorectal cancers than those with left-sided and rectal cancers. Wong et al. [15] reported that patients diagnosed with proximal cancers were 13 % less likely than those with distal cancers to survive in 5 years. Derwinger et al. [17] reported that right colon cancer resulted in a worse OS rate compared to left colon cancer, but not a worse CSS rate or disease-free survival rate. In contrast, Weiss et al. [8] reported no difference in mortality between right- and left-sided cancers for all stages combined. Relatedly, Powell et al. [10] showed no significant differences between tumor locations with regard to cancer-specific survival.

We found significant differences of OS and CSS only in patients with stage III CRC, but not for patients in stages I, II, and IV CRC. The 5-year OS rate of patients with stage III right-sided colon cancer was 21 % lower compared with the rate for those with stage III left-sided colon cancer. In addition, the 5-year CSS rate of patients with stage III right-sided colon cancer was 25 % lower than the rate for those with stage III left-sided colon cancer. The two differences were more prominent in patients with stage III disease than they were for all of the stages combined. Meguid et al. [6] showed a 4.2 % increased mortality risk in patients with right-sided colon cancer compared with those with left-sided colon cancer. They also reported that patients with stage III and IV disease right-sided colon cancer had a significantly increased mortality risk. However, patients with stage II disease right-sided colon cancer had a significanlyt decreased mortality risk. Benedix et al. [7] reported a 6 % lower 5-year OS rate in stage I disease and a 5 % lower OS rate in stage III disease in patients with right-sided colon cancer. However, they failed to show a significant difference in 5-year disease-free survival rates between patients with right- and left-sided colon cancer by sub-analysis with respect to various stages. Weiss et al. [8] reported no difference in mortality between right- and left-sided cancers for CRCs of all stages. However, they did report a lower mortality for stage II right-sided cancers specifically, in contrast with a higher mortality for stage III right-sided cancers.

In the present study, old age, poor differentiation, vascular invasion, perineural invasion, post-operative serum CEA level, and comorbidity of renal disease were demonstrated to be independent negative prognostic factors for OS and CSS in patients at all stages. Although tumor location was not found to be an independent prognostic factor, patients with right-sided cancers had a higher proportion of poorly differentiated tumors, a higher percentage of postoperative serum CEA levels ≧ 5 ng/ml, more locally advanced tumors and more stage II and III diseases. The similar results were also noted for patients with stage III CRC. Conversely, we also found no significant survival differences in stages I, II, and IV. The above results suggest that the factors that influence the prognosis of patients with CRC might be clinicopathologic characteristics other than tumor location. In addition, variations in tumor biology might be another potential factor related to the prognosis of patients with CRC [8]. For example, patients who are MSI-positive have a better OS and MSI status has been shown to be an independent favorable predictor of survival [8].

There are several limitations to the present study. First, the present study was a retrospective study of patients at only a single-institution. Second, for a retrospective study, the sample size was relatively small. Third, we did not examine the MSI statuses of the CRC patients studied; hence, any relevant information regarding the correlation between MSI status and the prognoses of patients with CRC is lacking.

## Conclusions

We have demonstrated that patients with right-sided colon cancer have poorer OS and CSS compared with those with left-sided colon cancer for all stages combined, with these survival differences being especially prominent in stage III disease. These survival differences may be the result of clinicopathologic characteristics, while aspects of tumor biology, such as MSI status, may be another crucial factor. Additional studies are needed to verify the relationship between tumor biology and prognosis for patients with CRC.

## Abbreviations

BMI: body mass index
CSS: cancer-specific survival
CRC: colorectal cancer
OS: overall survival
PFS: progression-free survival
CEA: Carcinoembryonic antigen
AJCC: American Joint Commission on Cancer
PD: Poorly differentiated
MD: Moderately differentiated
WD: Well differentiated
